# Overcoming resistance to arginine deprivation therapy using GC7 in pleural mesothelioma

**DOI:** 10.1016/j.isci.2024.111525

**Published:** 2024-12-02

**Authors:** Josephine Carpentier, Marta Freitas, Valle Morales, Katiuscia Bianchi, John Bomalaski, Peter Szlosarek, Sarah A. Martin

**Affiliations:** 1 Centre for Cancer Cell & Molecular Biology, Barts Cancer Institute, Queen Mary University of London, Charterhouse Square, London EC1M 6BQ, UK; 2 Polaris Pharmaceuticals, Inc., San Diego, CA 92121, USA; 3 Centre for Biomarkers & Biotherapeutics, Barts Cancer Institute, Queen Mary University of London, Charterhouse Square, London EC1M 6BQ, UK

**Keywords:** Natural sciences, Biological sciences, Systems biology, Cancer systems biology, Cancer

## Abstract

Pleural mesothelioma is a highly chemotherapy-resistant cancer. Approximately 50% of mesotheliomas do not express argininosuccinate synthetase 1 (ASS1), the rate-limiting enzyme in arginine biosynthesis, making arginine depletion with pegylated arginine deiminase (ADI-PEG20) an attractive therapeutic strategy. We investigated whether combinatory treatment composed of ADI-PEG20 and polyamine inhibitors constitutes a promising novel therapeutic strategy to overcome ADI-PEG20 resistance in mesothelioma patients. Treatment of ADI-PEG20-resistant cell lines with a range of different polyamine inhibitors demonstrated that ADI-PEG20-resistant cell lines were highly sensitive to the spermidine-analog GC7. We observed a synergistic effect of GC7 and ADI-PEG20 in both ADI-PEG20-sensitive and ADI-PEG20-resistant cell lines. Metabolomic analysis revealed that sensitivity to GC7 is due to inhibition of the Tricarboxylic (TCA) cycle. Significantly, combination of GC7 and ADI-PEG20 prevented the emergence of resistant cells *in vitro*. Taken together, we have identified the therapeutic potential of combinatorial treatment of ADI-PEG20 with GC7 for mesothelioma management.

## Highlights

• ADI-PEG20-resistant mesothelioma cells are highly sensitive to the spermidine-analog GC7
• Combinational treatment of GC7 and ADI-PEG20 is synergistic
• Sensitivity of ADI-PEG20-resistant cells to GC7 is due to inhibition of the TCA cycle
• Combinational treatment of GC7 and ADI-PEG20 prevented the emergence of resistant cells

## Graphical abstract

**Figure:**
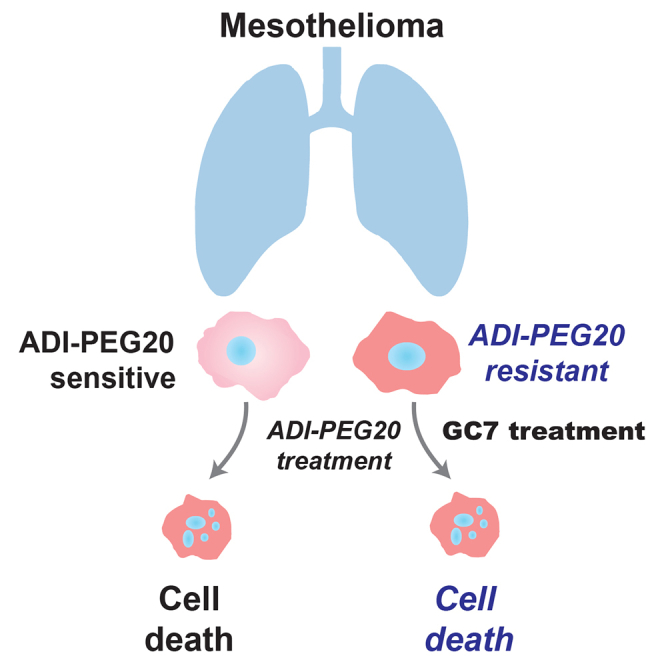

## Introduction

The prognosis for patients with the asbestos-related disease, pleural mesothelioma (PM) remains poor. PM is a highly chemotherapy-resistant disease with a median survival of 18 months with standard treatment.^1^ Treatment for advanced unresectable PM includes platinum-based chemotherapy in combination with pemetrexed and ipilimumab-nivolumab.^1^ Furthermore, recent studies support the combination of platinum-pemetrexed with pembrolizumab.^2^ A novel strategy based on arginine deprivation selectively kills mesothelioma cells that lack argininosuccinate synthetase 1 (ASS1), the rate-limiting enzyme in arginine biosynthesis.^3^ Approximately 50% of mesotheliomas do not express ASS1,^4^ making arginine depletion with pegylated arginine deiminase (ADI-PEG20) an attractive personalized therapeutic strategy^3^ that has shown significant activity in the randomized phase 2 Arginine Deiminase and Mesothelioma (ADAM) trial.^5^ Encouragingly, this trial achieved its primary endpoint of a significant improvement in progression-free survival above best supportive care. Furthermore, ADI-PEG20 in combination with platinum and pemetrexed (Tumours requiring arginine to assess pemetrexed and cisplatin (TRAP) Trial) showed a 100% disease control rate, leading to the ADI-PEG20 Targeting of Malignancies Induces Cytoxicity-Mesothelioma (ATOMIC-Meso) trial, which reported positive survival results for ADI-PEG20 in a phase 3 setting in non-epithelioid PM.^6^^,^^7^^,^^8^ Despite these promising initial results, resistance to ADI-PEG20 is a clinical obstacle. Understanding the mechanism of resistance and finding a way to overcome it remain a challenge, but it is necessary for ADI-PEG20 therapy to improve long-term outcomes for patients with ASS1- deficient cancer. To date, a number of mechanisms have been described for ADI-PEG20 resistance including ASS1 re-expression, antigenicity of the drug itself, and increased autophagy, and tumor-associated stromal cells may also provide arginine to ASS1-deficient tumor cells to counter the effect of ADI-PEG20.^9^^,^^10^^,^^11^^,^^12^^,^^13^ More recently, it has been shown that ASS1-independent tumor resistance to ADI-PEG20 can rely on macropinocytosis of extracellular vesicles that are degraded and recycled to supply arginine.^14^

Previously, we have generated the first model of ADI-PEG20 resistance in PM cells whereby we observed demethylation of the *ASS1* promoter, allowing re-expression of the ASS1 transcript and protein.^15^ Resistance was accompanied by decreased levels of acetylated polyamine metabolites in ASS1-deficient cells, indicative of reduced catabolism, together with a compensatory increase in expression of polyamine biosynthetic enzymes. Furthermore, this metabolic reprogramming revealed a synthetic lethal interaction between ASS1 loss and polyamine metabolism. In this study, we have revealed that a combined treatment of ASS1-deficient PM cells with ADI-PEG20 and the polyamine inhibitor GC7 not only kills ADI-PEG20-resistant cells but can also abrogate the emergence of ADI-PEG20-resistant cells.

## Results

### Polyamine metabolism is deregulated upon ADI-PEG20 resistance, irrespective of PM subtype

**Figure 1:**
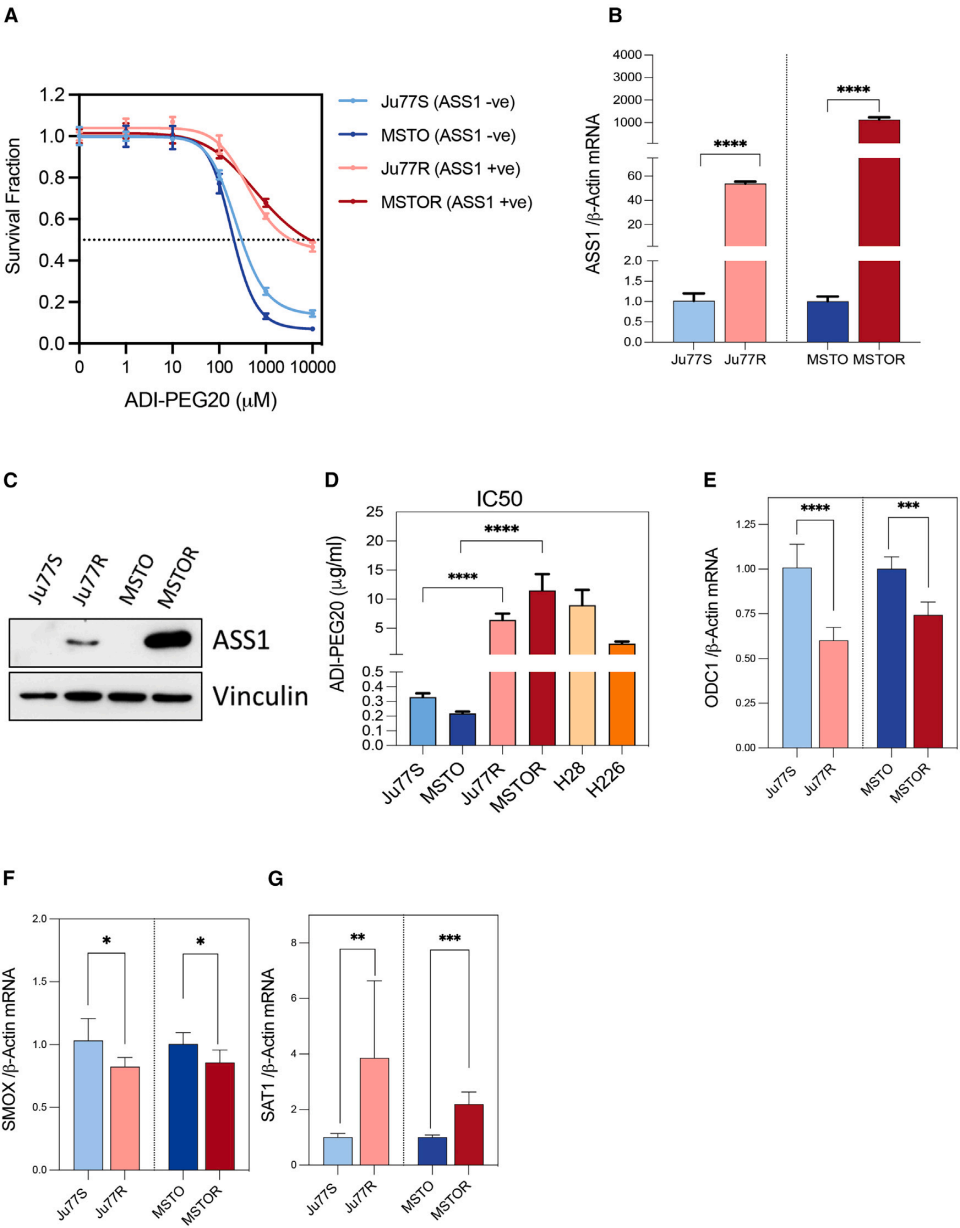
Polyamine metabolism is deregulated upon ADI-PEG20-resistance, irrespective of PM subtype (A) The ADI-PEG20-sensitive Ju77S and MSTO cells and the ADI-PEG20-resistant Ju77R and MSTOR MPM cells were treated with increasing concentrations of ADI-PEG20 (0, 1, 10, 100, 1,000, and 10,000 ng/mL). After 72 h treatment, cell viability was measured using an ATP-based luminescence assay. Data are represented as mean ± SEM. (B) Quantitative RT-PCR analysis of RNA extracted from Ju77S, Ju77R, MSTO, and MSTOR cells. mRNA expression was measured using ASS1 and β-actin Taqman probes. β-actin was used as a control. ∗∗∗∗*p* < 0.0005. Data are represented as mean ± SEM. (C) Western blot analysis of protein extracted from Ju77S, Ju77R, MSTO, and MSTOR cells. Protein expression was analyzed using anti-ASS1 and anti-Vinculin antibodies. Vinculin is used as a loading control. (D) IC50 values were calculated using the non-linear regression of the survival curve shown in Figure S1A. ∗∗∗∗*p* < 0.0005. Data are represented as mean ± SEM. (E–G) Quantitative RT-PCR analysis of RNA extracted from Ju77S, Ju77R, MSTO, and MSTOR cells. mRNA expression was measured using ODC1 (E), SMOX (F), SAT1 (G), and β-actin Taqman probes. β-actin was used as a control. ∗*p* < 0.05; ∗∗*p* < 0.005; ∗∗∗*p* < 0.0005; ∗∗∗∗*p* < 0.0005. Data are represented as mean ± SEM.

We previously generated a cell model of ADI-PEG20 resistance in the epithelial PM cell line Ju77, called Ju77R.^15^ To further understand ADI-PEG20 resistance across different PM subtypes, we generated a second ADI-PEG20-resistant cell line from the ASS1-negative (ASS1-ve) biphasic PM cell line, MSTO. MSTO cells were incubated with increasing concentrations of ADI-PEG20 (from 10 ng/mL to 1,000 ng/mL) until resistant cells emerged and proliferated (MSTO-Resistant (MSTOR); Figure 1A). We next compared the mRNA and protein expression of ASS1 in the two ADI-PEG20-resistant cell lines (Ju77R and MSTOR) and their parental cell lines (Ju77S and MSTO). Reverse-transcription PCR (RT-PCR) analysis revealed that Ju77R cells had a 50-fold increase in ASS1 RNA expression compared to the parental Ju77S cells, whereas MSTOR had a ∼1,000-fold increase in ASS1 mRNA expression compared to the MSTO cells (Figure 1B). Western blot analysis demonstrated a greater increase in ASS1 expression in MSTOR cells, in comparison to the Ju77R (Figure 1C). Our data suggest that a greater increase in ASS1 expression was induced upon ADI-PEG20 resistance in the MSTOR cells, in comparison to the Ju77R cells, therefore suggesting that these cell pairs may exhibit different or greater molecular differences following the generation of ADI-PEG20 resistance.

To determine the differential effect of ADI-PEG20 treatment on the viability of our ADI-PEG20-sensitive and ADI-PEG20-resistant cells, we treated the Ju77S, Ju77R, MSTO, and MSTOR, in addition to the ASS1-positive (ASS1+ve) PM cells, H28 and H226, with increasing concentrations of ADI-PEG20 (0.001, 0.01, 0.1, 1, or 10 μg/mL) for 72 h (Figure S1A). We observed that the viability of the two ASS1-ve (Ju77S, MSTO) were significantly reduced upon ADI-PEG20 treatment, whereas the four ASS1+ve cell lines (Ju77R, MSTOR, H28, H226) showed considerably less reduction in cell viability upon ADI-PEG20 treatment even at high concentrations, with IC50 values of <0.4 μg/mL ADI-PEG20 in the ASS1-ve cell lines and IC50 (half maximal inhibitory concentration) values of >2 μg/mL ADI-PEG20 in the ADI-PEG20-resistant and ASS1+ve cell lines (Figure 1D).

Previously, we observed that ADI-PEG20-resistant Ju77R cells have a deregulated polyamine metabolic pathway with a significant decrease in the expression of a number of enzymes involved in polyamine metabolism.^15^ We therefore wanted to determine whether similar changes in polyamine enzyme expression were occurring in the MSTOR cells upon ADI-PEG20 resistance. Significantly, we observed a decrease in the expression of the polyamine enzymes, ornithine decarboxylase (ODC1; Figure 1E) and spermine oxidase (SMOX; Figure 1F), and an increase in the levels of the key enzyme responsible for polyamine acetylation, spermidine/spermine N^1^-acetyltransferase 1 (SAT1; Figure 1G), in both the Ju77R and MSTOR cell lines, in comparison to their parental cells, Ju77S and MSTO, respectively. Therefore, our data confirm that ADI-PEG20 resistance is associated with a deregulation of the polyamine metabolic pathway, regardless of PM subtype, that may be potentially therapeutically targeted.

### GC7 prevents resistance to ADI-PEG20 treatment

**Figure 2:**
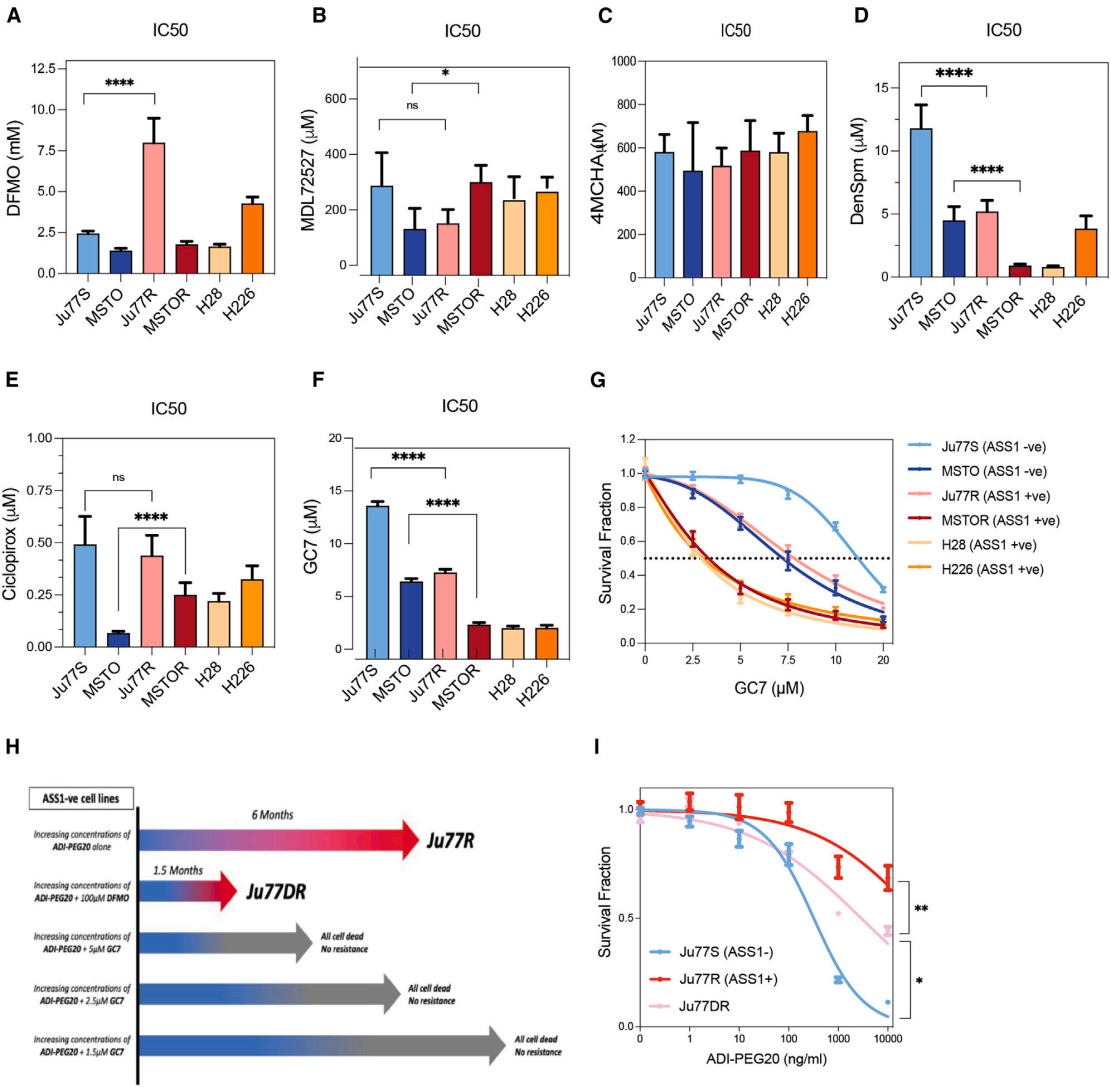
GC7 treatment prevents the generation of ADI-PEG20-resistant cells (A–F) The ADI-PEG20-sensitive Ju77S and MSTO cells and the ADI-PEG20-resistant Ju77R and MSTOR PM cells were treated with increasing concentrations of (A) DFMO, (B) MDL72527, (C) 4MCHA, (D) DenSpm, (E) Cicloprox, and (F) GC7. After 72 h treatment, cell viability was measured using an ATP-based luminescence assay. IC50 values were calculated using the non-linear regression of the survival curves shown in Figures S2A–S2F. ∗*p* < 0.05; ∗∗∗∗*p* < 0.0005; ∗∗∗∗∗*p* < 0.00005; ns, non-significant. Data are represented as mean ± SEM. (G) The ASS1-ve Ju77S and MSTO cells and the ASS1+ve Ju77R, MSTOR, H28, and H226 PM cells were treated with increasing concentrations of GC7 (0, 2.5, 5, 7.5, 10, and 20 μM). After 72 h treatment, cell viability was measured using an ATP-based luminescence assay. Data are represented as mean ± SEM. (H) Ju77S cells were cultured with ADI-PEG20 with or without different concentrations of either DFMO (100 μM) or GC7 (1.5, 2.5, and 5 μM). The capacity of Ju77S to generate resistant cells and how long before resistant cells appeared in the different conditions were evaluated and represented as a timeline. Ju77R represent cells that became resistant to ADI-PEG20 alone, after 6 months of treatment. Ju77DR represent cells that became resistant to ADI-PEG20 in combination with DFMO, after 1.5 months of treatment. (I) The Ju77S, Ju77R, and Ju77DR cells were treated with increasing concentrations of ADI-PEG20 (0, 1, 10, 100, 1,000, and 10,000 ng/mL). After 72 h treatment, cell viability was measured using an ATP-based luminescence assay. ∗*p* < 0.05; ∗∗*p* < 0.005. Data are represented as mean ± SEM.

We observed a significant rewiring of the polyamine metabolism pathway in both ADI-PEG20-resistant models (Feun et al.^11^; Figures 1E–1G). Therefore, we hypothesized that targeting polyamine metabolism could potentially provide a means of overcoming ADI-PEG20 resistance in PM patients. To investigate this, we treated our panel of ASS1-deficient (Ju7SS, MSTO), ASS1-proficient (H28, H226), and ADI-PEG20-resistant (Ju77R, MSTOR) PM cell lines with a panel of different polyamine-targeted agents to assess their potential for a combination with ADI-PEG20 (Figure S1B). These included the ODC1 inhibitor, α-difluoromethylornithine (DFMO); the spermidine synthase (SRM) inhibitor, *trans*-4-methylcyclohexylamine (4MCHA); the SMOX inhibitor MDL72527; the SAT1 activator, N1, N11-diethylnorspermine (DENSPM); the deoxyhypusine synthase (DHS) inhibitor, N^1^-guanyl-1,7-diaminoheptane (GC7); and the deoxyhypusine hydroxylase (DOHH) inhibitor, ciclopirox (CPX). By selecting these molecules, we were targeting different parts of the polyamine metabolic pathway including polyamine synthesis (DFMO, 4MCHA), polyamine catabolism (DENSPM, MDL72527), or the hypusination of eukaryotic initiation factor 5A (eiF5a) by spermidine (GC7, CPX) with the aim of gaining an overview of the effect of the modulation of the polyamine metabolic pathway on our panel of ADI-PEG20-sensitive and ADI-PEG20-resistant cell models. For each molecule, we analyzed their effect on cell viability after treatment in our panel of PM cell models (ASS1-ve, ADI-PEG20-Resistant, and ASS1+ve). To this end, Ju77S, Ju77R, MSTO, MSTOR, H28, and H226 cells were treated with increasing concentrations of each compound for 72 h. Cell viability was then analyzed (Figures S2A–S2F), and IC50 values were calculated (Figures 2A–2F). This analysis revealed that many of the polyamine-targeted agents reduced the cell viability of either the ADI-PEG20-resistant cells or the ASS1-ve cells but GC7 showed the most consistent significant inhibitory effect on ADI-PEG20-resistant cells, in comparison to their matched sensitive cell lines (Figures 2F and 2G). In addition, we observed inhibition of Ju77S cell viability, in comparison to Ju77R, upon DFMO treatment, validating our previous study that identified ODC1 inhibition was synthetically lethal with ASS1 loss in Ju77 cells (Figure 2A;^15^).

Therefore, we selected DFMO and GC7, to be used as potential combination treatments with ADI-PEG20, with the aim of overcoming resistance to arginine deprivation therapy in mesothelioma. To determine whether a combination of either of these compounds with ADI-PEG20 could prevent or delay the generation of ADI-PEG20-resistant cells, Ju77S cells were incubated with increasing concentrations of ADI-PEG20 (from 10 ng/mL to 1,000 ng/mL) in combination with DFMO (100 μM) or GC7 (1.5, 2.5, or 5 μM) until resistant cells emerged and proliferated. Unexpectedly, our results showed that not only did DFMO treatment in combination with ADI-PEG20 not prevent or delay the generation of resistance but also resistant cells emerged significantly faster than when treated with ADI-PEG20 alone (1.5 vs. 6 months; Figures 2H and 2I). Interestingly, treating Ju77S cells with GC7 in combination with ADI-PEG20 prevented the emergence of ADI-PEG20-resistant cells, such that we never observed any ADI-PEG20-resistant cell clones emerge, when the cells were grown in the presence of GC7 with ADI-PEG20. To fully establish whether this was true, we performed the experiment with different concentrations of GC7, and regardless of GC7 concentration we never observed any resistant cells emerge in the combination treatment (Figure 2H). These results highlight the potential of GC7 as a combination therapy with ADI-PEG20 that may prevent the generation of ADI-PEG20-resistant cells.

### Combined ADI-PEG20 and GC7 treatment decreases cell viability and proliferation in 3D spheroid models

**Figure 3:**
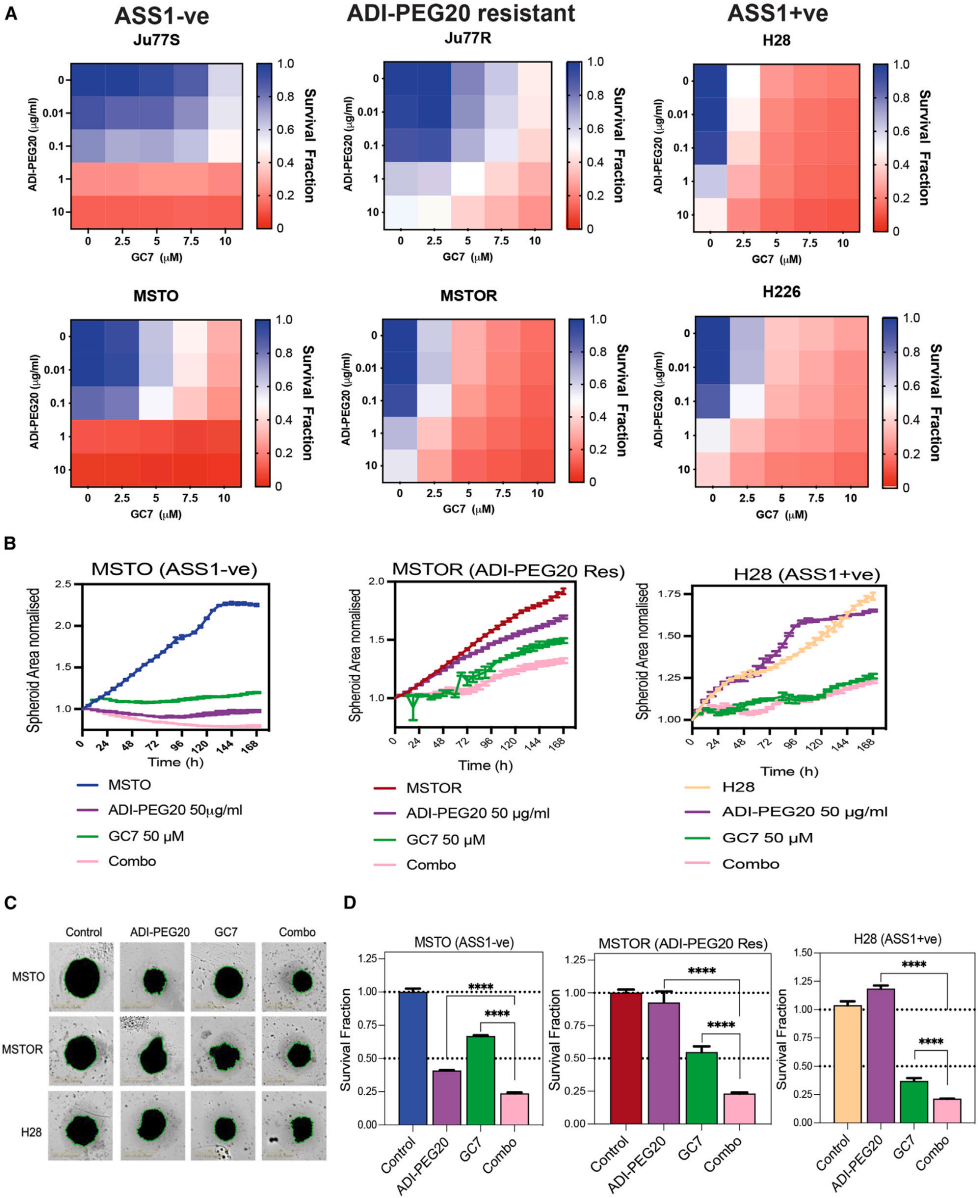
Combined ADI-PEG20 and GC7 treatment decreases cell viability and proliferation in 3D spheroid models (A) A panel of ASS1-ve (Ju77S, MSTO), ASS1+ve, ADI-PEG20-resistant (Ju77R, MSTOR), and ASS1+ve (H28, H226) cells were treated either 0.001, 0.01, 0.1, 1, or 10 μg/mL of ADI-PEG20 and/or 0, 2.5, 5, 7.5, or 10 μM GC7, for 72 h. Cell viability was measured using the CTG assay. Survival fractions were normalized to luminescent values of the untreated cells. (B) A panel of ASS1-ve (MSTO), ADI-PEG20-resistant (MSTOR), and ASS1+ve (H28) cells were cultured in low-attachment plates for 4 days to form 3D spheroids. Upon spheroid formation, cells were treated with ADI-PEG20 (50 μg/mL) or GC7 (50 μM) for 7 days. Spheroid area upon treatment was measured using the IncuCyte live-cell imaging system overtime. The spheroids were imaged every 6 h, and their size was normalized back to the size of each spheroid at t = 0 h. (C) Representative images of spheroids after 7 days of treatment with 50 μg/mL ADI-PEG20 and or 50 μM GC7, alone or in combination (Combo). Green outline of the spheroids corresponds to the detection of the area of the sphere by the IncuCyte imaging software. (D) Spheroid viability of MSTO, MSTOR, and H28 cells upon treatment with ADI-PEG20 (50 μg/mL) or GC7 (50 μM) for 7 days was measured using the CTG assay. Luminescence was measured at the end of the treatment, and viability readings were normalized back to the untreated spheroid viability. ∗∗∗∗*p* < 0.0001. Data are represented as mean ± SEM.

To further investigate the consequences of combined ADI-PEG20 and GC7 treatment in our panel of PM cell lines, we treated the ASS1-ve, ASS1+ve, and ADI-PEG20-resistant cells for 72 h with increasing concentrations of ADI-PEG20 (0.01, 0.1, 1, and 10 μg/mL) and/or GC7 (2.5, 5, 7.5, and 10 μM). Cell viability was then measured with CellTiter-Glo (CTG), an ATP-based luminescent assay (Figure 3A). We observed a significant reduction in cell viability in the ASS1-ve cell lines upon ADI-PEG20 treatment alone, as expected, with little inhibitory effect on the ASS1+ve and ADI-PEG20-resistant cell lines. In contrast, treatment with GC7 was significantly more cytotoxic in the ADI-PEG20-resistant, ASS1-expressing cell lines. Both the ASS1+ve cell lines, H28 and H226, were also sensitive to GC7. Significantly, a reduction in cell viability was observed in all cell lines, upon treatment with the combination of ADI-PEG20 (1 μg/mL) and GC7 (7.5 μM; Figure 3A). To confirm that the effects on cell viability upon ADI-PEG20 and GC7 treatment were caused by a decreased number of viable cells and not because of a modulation of the cellular ATP level, a cell proliferation assay was performed over time upon treatment of ADI-PEG20 and/or GC7 in all our cell models using the IncuCyte live-cell imaging system (Figures S3A–S3F). Our analysis confirmed a similar increased inhibitory effect of the combination treatment of ADI-PEG20 and GC7, compared to the use of each compound alone, on the proliferation of all ASS1-ve, ASS1+ve, and ADI-PEG20-resistant cell lines over time.

Upon validation of the combination of ADI-PEG20 and GC7 in a 2D cell culture model, we next aimed to understand the effect of the combination of ADI-PEG20 and GC7 treatment on 3D spheroid models, generated with different PM cell lines (MSTO, MSTOR, H28) (Figures 3C and 3D). We seeded MSTO (ASS1-ve), MSTOR (ADI-PEG20-resistant), and H28 (ASS1+ve) cells on low-attachment 96-well plates and cultured cells for 4 days until 3D spheroids were formed After spheroid formation, spheres were treated with or without ADI-PEG20 and/or GC7 for 7 days. The IncuCyte live-cell imaging system was used to measure the size of the spheroids every 6 h for 7 days (Figures 3B and 3C), and a CTG assay was used to assess the viability of the spheres at the end of treatment (Figure 3D). In the vehicle-treated cells, we observed increased spheroid volume overtime, as expected in all cell lines (Figure 3B). Upon ADI-PEG20 treatment, we observed that the volume of the ASS1-ve, MSTO spheroids did not increase overtime in comparison to the initial time point, validating that ADI-PEG20 was inhibiting the size of the sphere over 7 days of treatment (Figure 3B). Upon GC7 treatment, we observed reduced sphere volume, upon comparison to the vehicle-treated spheres for each cell line. Upon treatment with the combination of ADI-PEG20 and GC7, the sphere size was reduced in all cell lines in comparison to either drug alone, validating the inhibitory effect of the drug combination. To confirm that the reduction in spheroid size was due to a decreased number of viable cells and not a higher sphere density or aggregation, the CTG cell viability assay was performed on the spheroids. Upon combination treatment, the survival fraction of the spheroid cells after the drug combination was significantly less than that after either of the drugs alone (Figure 3D). These data suggest that, in both 2D and 3D cellular models, the combination of GC7 and ADI-PEG20 could also induce an inhibitory effect in PM cells. As it has been shown that 3D spheroid models are more reflective of the phenotype of tumors *in vivo*, our results highlight the potential of a combined treatment of ADI-PEG20 and GC7 for the treatment of ASS1-ve, ASS1+ve, and ADI-PEG20-resistant PM cells.

### GC7 treatment is synergistic with ADI-PEG20 *in vitro* and *in vivo*

**Figure 4:**
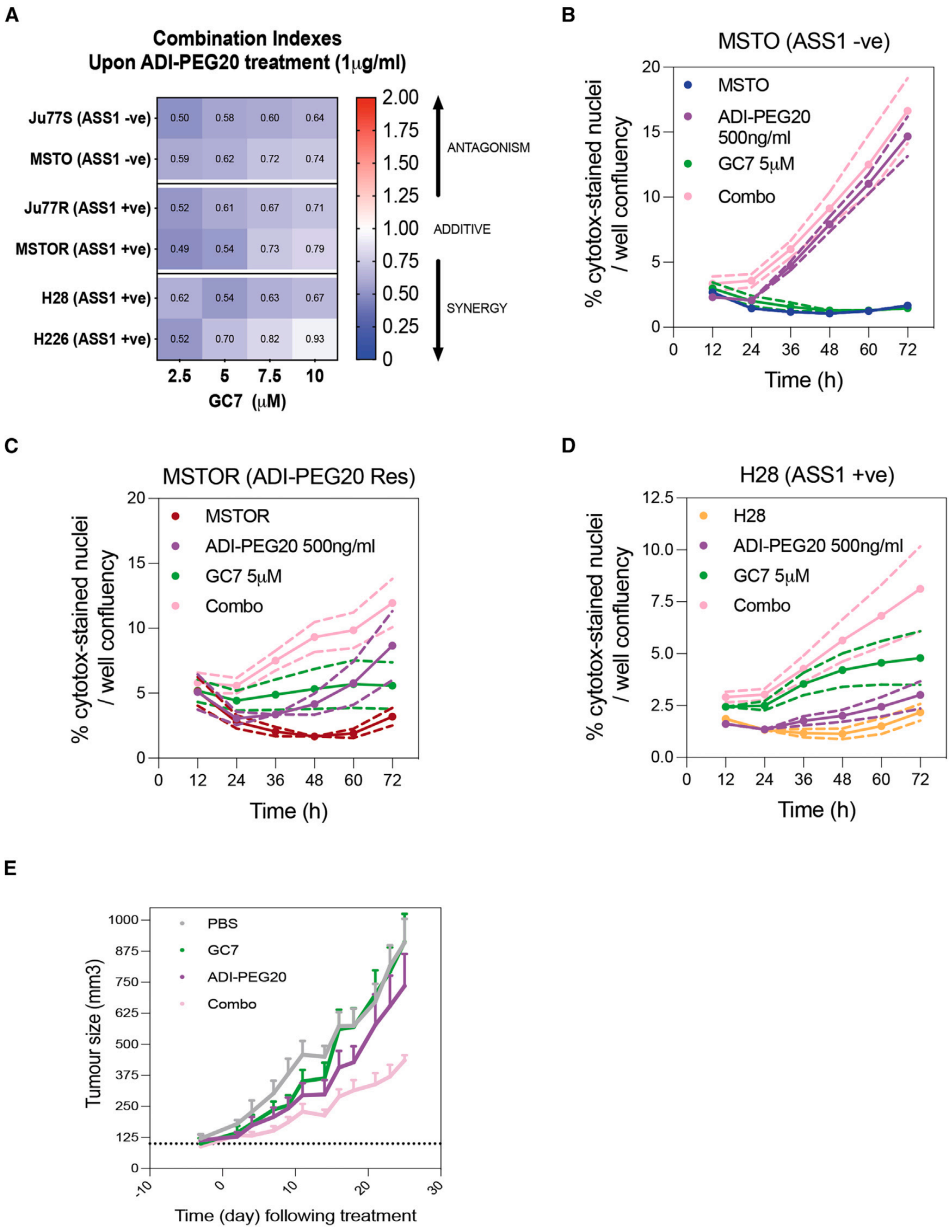
Combination treatment of ADI-PEG20 and GC7 is synergistic in PM cells (A–D) (A) A panel of ASS1-ve (Ju77S, MSTO), ADI-PEG20-resistant (Ju77R, MSTOR), and ASS1+ve (H28, H226) cells were treated with escalating concentrations of ADI-PEG20 (0, 10, 100, 1,000, and 10,000 ng/mL), escalating concentrations of GC7 (0, 2.5, 5, 7.5, and 10 μM), or a combination of all concentrations used. After 72 h of treatment, cell viability was evaluated using a CTG assay. Survival fractions were normalized to luminescent values of the untreated cells. Combination indexes of the combination of ADI-PEG20 and GC7 were calculated using the CalcuSyn software. Data shown in each cell of the heatmap correspond to the median combination index of 1 μg/mL ADI-PEG20 in combination with escalating concentrations of GC7 (0, 2.5, 5, 7.5, and 10 μM) as calculated by the CalcuSyn software. Combination index data for all concentrations of ADI-PEG20 are shown in Figure S4. The MSTO (B), MSTOR (C), and H28 (D) Cells were cultured overtime with or without ADI-PEG20 (500 ng/mL) and GC7 (5 μM) alone or in combination (Combo). Cells were stained with the IncuCyte Cytotox Green dye to evaluate the number of cytotoxic cells overtime following treatment. Images were taken every 2 h over a period of 3 days. Upon membrane permeabilization, nuclei were stained green by the dye, indicating cytotoxicity. The fraction of cytotoxic cells were normalized to the total cell confluency using the IncuCyte live-cell imaging system. A confluency mask was applied to each individual image and normalized to the first image (t = 0 h). (E) *In vivo* efficacy experiments were performed on NOD-SCID mice injected with MSTO cells (1.6 × 10^6^ cells). When the tumors were measurable, mice were treated 2 times a week by intraperitoneal injection with 10 mg/kg GC7, 5 IU ADI-PEG20 alone or in combination (Combo), or vehicle (PBS). Tumors were measured three times a week and normalized to initial treatment measurements. Data are represented as mean ± SEM.

We observed reduced cell viability (Figure 3A) and proliferation (Figure 3B) following treatment with the combination of ADI-PEG20 with GC7; therefore, we next investigated whether combining ADI-PEG20 with GC7 would have a synergistic effect on cells, compared to the effect of the drugs alone. Using the CalcuSyn software, we calculated the combination index for each different concentration of drug used in the drug combination. A combination index value between 0.9 and 1.1 reflects an additive effect, while values below 0.9 reflect synergy and, alternatively, a value above 1.1 reflects an antagonistic effect. ASS1-ve cells lines (Ju77S, MSTO), ADI-PEG20-resistant cell lines (Ju77R, MSTOR), and ASS1+ve cell lines (H28, H226) were treated as before, with increasing concentrations of ADI-PEG20 (0.01, 0.1, 1, and 10 μg/mL) alone or in combination with GC7 (2.5, 5, 7.5, and 10 μM). After 72 h, cell viability was assessed using the CTG assay, and the fraction of cells surviving the treatment was entered in the CalcuSyn software. A heatmap representing the combination indexes was generated (Figures 4A and S4A–S4F). Our analysis revealed that the combination of ADI-PEG20 and GC7 was synergistic at 1 μg/mL ADI-PEG20 and 2.5, 5, 7.5, and 10 μM GC7 in all cell lines (Figure 4A). Therefore, we have identified a novel synergistic interaction upon treatment with GC7 and ADI-PEG20 in ASS1-ve, ASS1+ve, and ADI-PEG20-resistant PM cell lines.

To determine whether the combination of ADI-PEG20 and GC7 was merely slowing down cell viability and proliferation, or whether it was causing a cytotoxic effect on the cells, we performed a cytotoxicity assay using the IncuCyte Cytotox Green dye, which is a marker of cell membrane permeabilization (Figures 4B–4D). Therefore, if a nucleus stained green with the dye, it would suggest that the cell membrane was permeabilized upon treatment and indicate a cytotoxic effect on the cells. To this end, MSTO, MSTOR, and H28 cells were treated with ADI-PEG20 (500 ng/mL) and/or GC7 (5 μM) over 3 days and monitored with the IncuCyte live-cell imaging system. Images were taken every 2 h, and confluency and green channel detection were measured. To determine the fraction of cytotoxic cells among the cell population, the amount of green-stained nucleus detected (cytotoxic cells) were normalized to the cell confluency for each time point. Our results showed that, upon treatment with ADI-PEG20, an increase in the fraction of cytotoxic cells was observed for MSTO cells with a ∼9-fold increase from control after 72 h treatment (Figure 4B); whereas ∼2.7- and ∼1.4-fold increases were observed for MSTOR cells (Figure 4C) and H28 cells (Figure 4D), respectively. These results confirmed the differences in sensitivity to ADI-PEG20 in ASS1-ve and ASS1+ve cell lines. Upon treatment with GC7, we observed that the fraction of cytotoxic cell was greater in the ASS1+ve cells lines, with an increase of ∼0.9-, ∼1.75-, and ∼2.2-fold for MSTO, MSTOR, and H28 cells. Interestingly, upon combination of ADI-PEG20 and GC7, we observed a significant increase in cell cytotoxicity in all three cell lines compared to the cytotoxicity observed by the drugs alone. These data suggested that a combination of ADI-PEG20 and GC7 caused a significant increase in cytotoxicity in our cell line panel, compared to the use of the individual drugs, and confirmed the synergistic effect we previously observed.

To examine the *in vivo* efficacy of a combination of ADI-PEG20 and GC7, the ASS1-deficient MSTO cells were injected subcutaneously into non-obese diabetic (NOD)- severe combined immunodeficiency (SCID) mice and xenografted mice were subjected to treatment 2 times a week with vehicle (PBS), GC7, ADI-PEG20, or combined GC7-ADI-PEG20 treatment (Figure 4E). We observed that tumor growth was significantly reduced (*p* = 0.0005) by combined GC7-ADI-PEG20 treatment when compared to vehicle or either drug alone. Taken together, these *in vivo* observations further indicate that combined GC7-ADI-PEG20 treatment has potential clinical utility in preventing ADI-PEG20 resistance for ASS1-deficient MPM patients, for which ADI-PEG20 resistance is a critical issue.

### Reduced TCA cycle metabolites upon combined ADI-PEG20 and GC7 treatment

**Figure 5:**
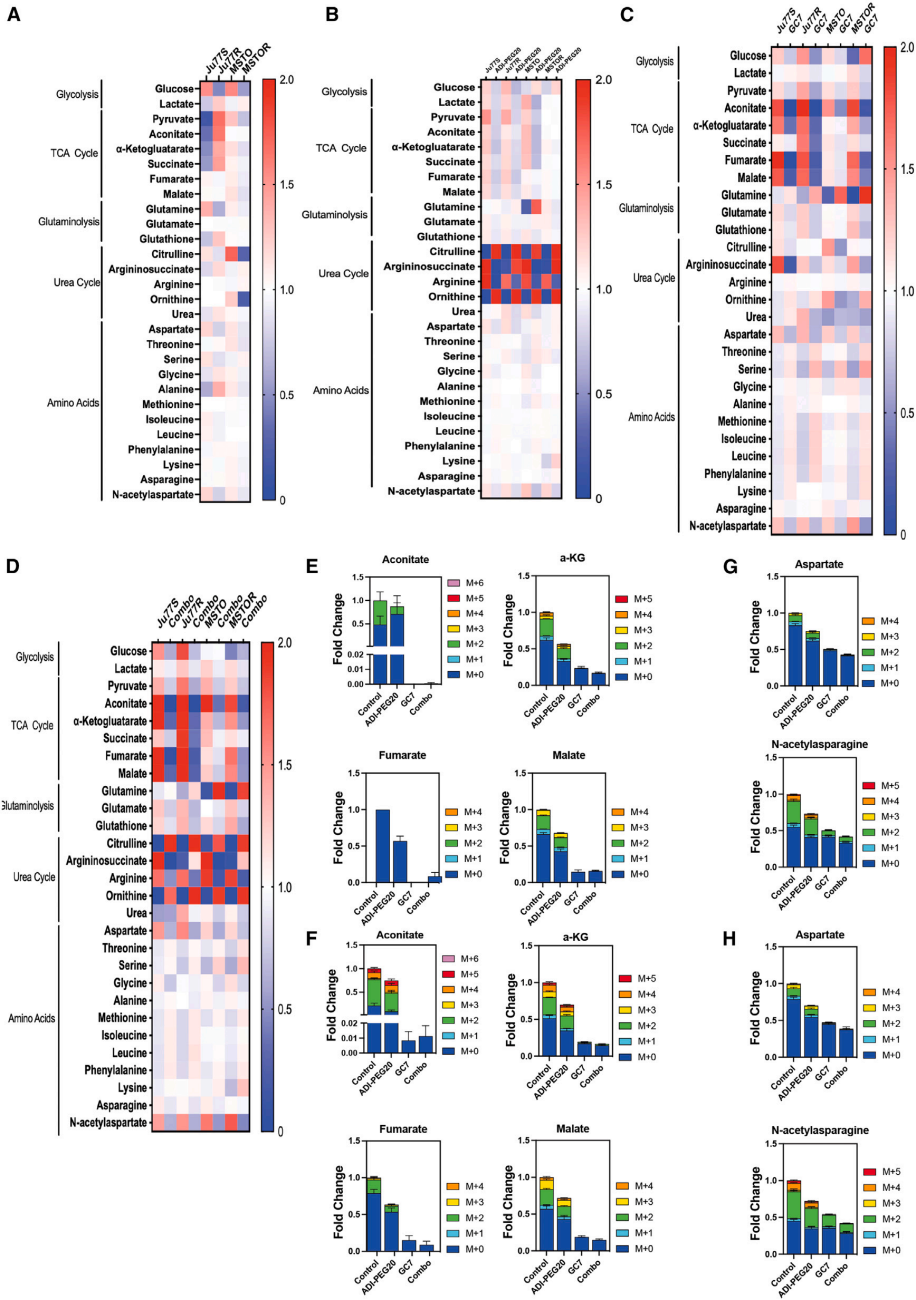
GC7 treatment causes reduced TCA cycle metabolites in PM cells A panel of ASS1-ve (Ju77S, MSTO) and ADI-PEG20-resistant (Ju77R, MSTOR) cell lines were cultured for 24 h with or without 500 ng/mL ADI-PEG20 and/or 5 μM GC7 before being harvested for metabolite extraction. Samples were processed by LC/MS for targeted metabolite detection. Each individual level has been normalized to the total ion content of the sample. Metabolite level changes of Ju77S, Ju77R, MSTO, and MSTOR (A), upon ADI-PEG20 (B), GC7 (C), and ADI-PEG20 and GC7 in combination (D) are shown as a heatmap displaying the *Z* scores of the variation of the expression of each metabolite. Ju77S (E and G) and Ju77R (F and H) cells were cultured for 24 h with or without 500 ng/mL ADI-PEG20 and/or 5 μM GC7 in RPMI-1640 with 2 g/L 13-C labeled glucose. Cells were harvested for metabolite detection. Each individual metabolite isotopomers have been normalized to the total ion content of the sample. Associated isotopomers are shown as a stacked bar chart. M+0, dark blue; M+1, light blue; M+2, green; M+3, yellow; M+4, orange; M+5, red; M+6, purple. Data are represented as mean ± SEM.

Our results demonstrate that a combined treatment of GC7 and ADI-PEG20 can eliminate the generation of drug-resistant PM cells. To understand the mechanism behind this, we performed targeted stable isotope tracing metabolomics on our panel of ADI-PEG20-sensitive and ADI-PEG20-resistant cell lines, incubated with either ADI-PEG20 or GC7, alone or in combination. Ju77S, Ju77R, MSTO, and MSTOR cells were incubated with 500 ng/mL ADI-PEG20 and/or GC7 for 24 h before extracting metabolites for a targeted metabolomics analysis of 30 steady-state metabolite levels belonging to different pathways including glycolysis, TCA cycle, urea cycle, glutaminolysis, and amino acids. Liquid chromatography-mass spectrometry (LC/MS) separation was performed, and data were normalized to the total sample ion content. We then used these data to calculate the fold change of each metabolite between the different conditions and evaluate their significance. Prior to treatment, the analysis of the 30 steady-state metabolite levels revealed that ADI-PEG20 resistance resulted in an increase in the total level of several different metabolites belonging to the glycolysis pathway and TCA cycle in the Ju77R cells (Figure 5A). It is of note that the MSTOR cells do not exhibit increased TCA metabolites. We hypothesized that this may be due to the fact that MSTOR cells demonstrate a significantly higher expression of ASS1 than Ju77R. Prolonged ADI-PEG20 treatment and subsequent ADI-PEG20 resistance may have also differently influenced the metabolism of these cells in terms of the TCA cycle metabolites. Future studies are required to understand specifically how ADI-PEG20 resistance may increase TCA cycle metabolites and how levels of ASS1 expression may influence this.

Following ADI-PEG20 treatment, we observed that the urea cycle was the most significantly affected pathway upon ADI-PEG20 treatment, in all our cell models (Figure 5B). ADI-PEG20 can convert arginine into citrulline, and as expected we observed a decrease in arginine and a consequential increase in citrulline upon ADI-PEG20 treatment, providing a positive control for the ADI-PEG20 treatment. In addition, the level of argininosuccinate was significantly decreased in the ASS1-ve Ju77S and MSTO cell lines after ADI-PEG20 treatment, whereas argininosuccinate levels increased in the ADI-PEG20-resistant Ju77R and MSTOR cell lines, correlating with ASS1 re-expression. Analysis of the data from cells treated with GC7 alone revealed that TCA cycle metabolites (aconitate, α-ketoglutarate, succinate, fumarate, and malate) were all significantly reduced upon GC7 treatment in all cell lines (Figure 5C). Metabolites derived from TCA cycle (aspartate, N-acetylaspartate, and argininosuccinate) were also significantly reduced upon GC7 treatment. This is in agreement with a previous study showing that GC7 treatment on macrophages results in reduced TCA cycle metabolites.^16^ Next, upon the combination of GC7 and ADI-PEG20 treatment, we observed a similar effect with the drug combination on the urea cycle as we observed upon ADI-PEG20 treatment alone (Figure 5D). Interestingly, the alterations observed on the TCA cycle induced by GC7 alone were also observed upon treatment with the combination of GC7 and ADI-PEG20.

Upon incubating with 13-C-labeled glucose the Ju77S (Figures 5E and 5G), Ju77R (Figures 5F and 5H), MSTO (Figures S5A and S5C), and MSTOR (Figures S5B and S5D) cells, we observed that the fractional contribution of glucose was significantly changed for the TCA cycle metabolites (aconitate, α-ketoglutarate, fumarate, and malate) and metabolites derived from the TCA cycle (aspartate, N-acetylaspartate), upon the combination treatment of ADI-PEG20 and GC7, similar to the changes observed upon treatment with GC7 alone, such that no significant differences were observed between the fractional contribution of glucose for these metabolites when comparing GC7 treatment and the combination treatment (Figure S5E). Consequently, the combination treatment resulted in an inhibition of the production of glucose-derived TCA cycle metabolites and derivatives, similar to GC7 alone. Overall, ADI-PEG20 and GC7 can significantly rewire cellular metabolism by inhibiting the synthesis of glucose-derived TCA cycle metabolites.

### GC7 targets ADI-PEG20-resistant cells by inhibition of the TCA cycle

**Figure 6:**
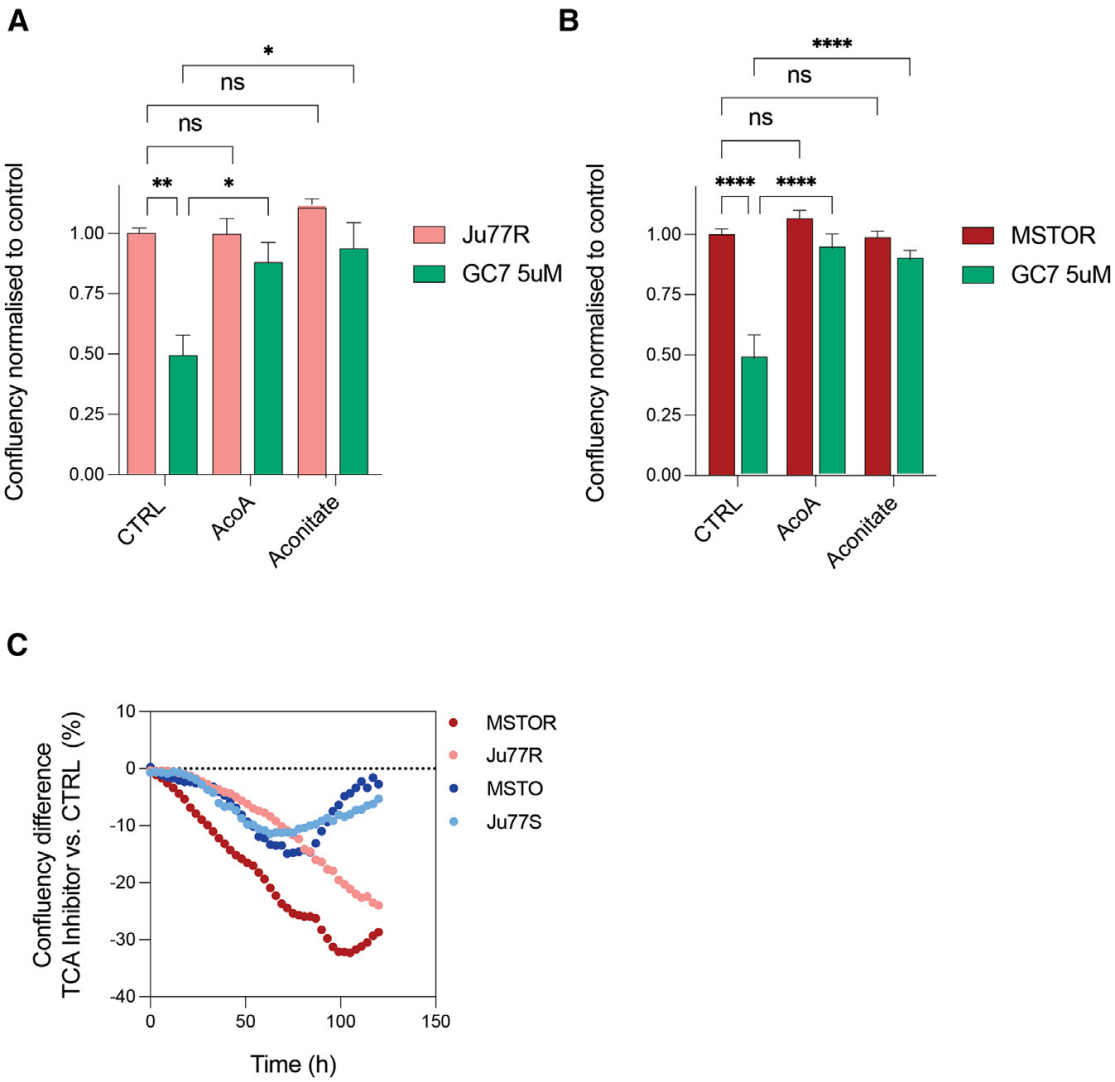
Inhibition of the TCA cycle by GC7 targets ADI-PEG20-resistant cells (A and B) The Ju77R (A) and MSTOR (B) cells were treated with control (CTRL; DMSO; 0.01%), GC7, acetyl-CoA (AcoA), and aconitate alone or in combination, and cell proliferation was analyzed using the IncuCyte live-cell imaging system. Images were taken every 3 h over a period of 3 days. A confluency mask was applied to each individual image and normalized to the first image (t = 0 h). ∗*p* < 0.05; ∗∗*p* < 0.005; ∗∗∗∗*p* < 0.0001. Data are represented as mean ± SEM. (C) The Ju77S, Ju77R, MSTO, and MSTOR cells were treated with either control (CTRL; DMSO; 0.01%) or the TCA cycle inhibitor, CPI-613. Cell proliferation was analyzed using the IncuCyte live-cell imaging system. Images were taken every 3 h over a period of 7 days. A confluency mask was applied to each individual image and normalized to the first image (t = 0 h).

Given our metabolomics analysis revealed that the ADI-PEG20-resistant Ju77R cells had increased levels of TCA cycle metabolites and treatment with GC7 alone or in combination significantly reduce TCA cycle metabolites, we hypothesized that perhaps ADI-PEG20-resistant cells are more dependent on the TCA cycle for survival. To understand whether GC7 was cytotoxic in our ADI-PEG20-resistant Ju77R and MSTOR cells due to TCA cycle inhibition, we treated the cells with either GC7 alone or in combination with the TCA metabolites, acetyl-coenzyme A (CoA) (AcoA), or aconitate and measured cell viability (Figures 6A and 6B). Significantly, we observed that addition of these TCA metabolites could rescue the cytotoxicity induced by GC7 treatment in ADI-PEG20-resistant cells, therefore strongly suggesting that GC7 is sensitizing ADI-PEG20-resistant cells via inhibition of the TCA cycle. To further investigate the role of the TCA cycle in ADI-PEG20 resistance, we treated our panel of cell lines with a different TCA cycle inhibitor, CPI-613. CPI-613 is a clinically available inhibitor of mitochondrial TCA, which targets pyruvate dehydrogenase and α-ketoglutarate dehydrogenase. We observed that, over time, ASS1-ve cells were initially inhibited by CPI-613 treatment but were able to recover whereas the ADI-PEG20-resistant cells remained inhibited following CPI-613 treatment (Figure 6C). Therefore, our data suggest that the cytotoxicity observed upon GC7 treatment in the ADI-PEG20-resistant cells is due to inhibition of the TCA cycle.

Taken together, our data indicate that a combination treatment of GC7 and ADI-PEG20 has significant potential clinical utility to minimize drug resistance in patients with ASS1-ve mesothelioma tumors, for which therapeutic options currently do not exist.

## Discussion

Our study reveals a novel interplay between arginine and polyamine metabolism that can potentially be exploited for the treatment of ADI-PEG20-resistant PM patients. To date, ADI-PEG20 has been indicated primarily as a potential treatment for ASS1-ve and arginine-auxotrophic cancers. Several clinical trials have used a threshold of 50% of ASS1-deficient cells in the tumor as one of the eligibility criteria for ADI-PEG20 treatment.^6^ These trials have highlighted the heterogeneous expression of ASS1 frequently observed suggesting that, even if the majority of tumor cells are ASS1-ve and therefore sensitive to arginine deprivation therapy, ASS1+ve cells may survive and proliferate upon ADI-PEG20 treatment. Our results indicate that combined ADI-PEG20 and GC7 treatment is synergistic in both ASS1-ve and ASS1+ve cells. Together these data suggest that combined GC7 and ADI-PEG20 treatment may be instrumental in overcoming the limitations of heterogeneous ASS1 expression for the treatment of PM patients.

We have previously reported that the ASS1-ve Ju77S cells were increasingly sensitive to DFMO treatment, and a synergistic effect of ADI-PEG20 and DFMO was observed in the Ju77S and Ju77R cells.^15^ Therefore, we hypothesized that combined ADI-PEG20 and DFMO treatment could be a potential way to overcome ADI-PEG20 resistance. However, upon combination of ADI-PEG20 with DFMO, the polyamine inhibitor did not prevent the appearance of ADI-PEG20 resistance overtime. Surprisingly, ADI-PEG20-resistant cells appeared faster upon treatment with the combination of ADI-PEG20 and DFMO compared to ADI-PEG20 alone. DFMO is an enzymatic inhibitor of ODC1, the enzyme catalyzing the conversion of ornithine into putrescine, and is considered as one of the rate-limiting enzymes of polyamine synthesis. We have shown that, upon ADI-PEG20 resistance, ODC1 expression was decreased in the resistant cell lines, compared to the parental ASS1-ve cell lines. Therefore, our data suggest that ADI-PEG20 treatment is associated with reduced ODC1 expression leading to ADI-PEG20 resistance. This is further supported by the rapid generation of ADI-PEG20 resistance when combined with DFMO, which occurs more rapidly than ADI-PEG20 alone. Future studies are required to understand how ODC1 expression may influence the expression of ASS1 ultimately governing the response to ADI-PEG20 treatment.

Recently targeting the polyamine-hypusine axis has been developed as a potential therapeutic target for cancer treatment. DHS, DOHH, and eiF5a inhibitors have been investigated for different cancer types *in vitro* and *in vivo.* GC7 is the hypusine-axis inhibitor that has been the most studied for cancer treatment and other pathologies.^17^^,^^18^^,^^19^^,^^20^ The first notable effect of GC7 was that it inhibited the growth of several cancer types both *in vitro* and *in vivo*.^21^^,^^22^^,^^23^^,^^24^^,^^25^^,^^26^^,^^27^^,^^28^^,^^29^ Significantly, Preukschas et al. suggested that, while GC7 significantly inhibited the proliferation of glioblastoma cell lines, normal human astrocytes were not sensitive to the drug, suggesting reduced toxicity to normal cells.^22^ Depending on the cancer type, different mechanisms of action of GC7 have been described. Some studies suggest a role for GC7 as an inhibitor of the epithelial-mesenchymal transition.^25^^,^^27^^,^^30^ GC7 treatment was also shown to suppress the intracellular level of reactive oxygen species in hepatocellular carcinoma (HCC) cells.^30^ Studies have shown that GC7 treatment can promote c-Myc translation^26^ and downregulate HIF-1α.^27^ Interestingly, while ASS1 loss is due to promoter methylation in MPM cells, reduced ASS1 expression has been shown to be mediated by c-Myc and HIF-1α in other cancers including melanoma.^31^ To fully elucidate the potential of ADI-PEG20-GC7 as a combination therapy in ASS1-ve cancers, it will be important to test this combination in a range of different cancers where mechanisms of ASS1 regulation differ.

Our findings reveal that upon GC7 treatment, the level of TCA cycle metabolites was greatly decreased in our cell models. This was shown to be linked to inhibition of the production of glucose-derived TCA cycle metabolites. Other reports also suggest an effect of GC7 toward mitochondrial metabolites.^16^^,^^19^ Melis et al. suggested that GC7 treatment induced silencing of mitochondria metabolism and prevented anoxic cell death, leading to improved kidney transplant outcome.^19^ Interestingly, GC7-induced mitochondrial inhibition was accompanied with an increase in glucose consumption in normal kidney cells. Puleston et al. showed that GC7 can regulate the expression of the TCA cycle enzymes and prevent oxidative phosphorylation (OxPhos)-dependent macrophage activation.^16^ Both studies showed a downregulation of OxPhos and O_2_ consumption due to reduced expression of complexes I, II, and III of the electron transport chain, upon inhibition of hypusine formation. Therefore, the precise mechanism behind GC7-mediated TCA cycle inhibition is still to be elucidated. Furthermore, treatment with an alternative polyamine-hypusine axis inhibitor, CPX (Figures 2E and S2E), or the specific inhibition of DHS with small interfering RNA (data not shown) did not overcome the resistance to ADI-PEG20. Therefore, taken together, our data suggest that GC7 may overcome ADI-PEG20 resistance and/or TCA inhibition via a different mechanism than DHS inhibition or inhibition of the polyamine-hypusine axis.

Taken together, we have elucidated a novel interplay between arginine and polyamine metabolism that can potentially be exploited for the treatment of ADI-PEG20-resistant MPM patients. Preclinical studies have previously shown that this therapeutic strategy is applicable to both non-epithelioid and epithelioid MPM. The clinical study, ATOMIC-Meso, focused on non-epithelioid MPM due to a 2- to 3-fold higher rate of arginine dependency compared to the epithelioid subtype. Collectively, our data suggest that treating ASS1-ve MPM tumors irrespective of subtype with a combination of ADI-PEG20 and GC7 may abrogate the emergence of resistant cells and can be exploited clinically for the treatment of these patients.

### Limitations of the study

Our study has some limitations that are necessary to acknowledge. Given that DHS inhibition did not overcome the resistance to ADI-PEG20, it remains unclear how GC7 can overcome ADI-PEG20 resistance. In addition, although we did not observe any ADI-PEG20-resistant clones emerging upon combinatorial treatment of ADI-PEG20 and GC7, we cannot rule out this may be due to experimental limitations.

## Resource availability

### Lead contact

Further information and requests for resources and reagents should be directed to and will be fulfilled by the lead contact, Sarah A. Martin (sarah.martin@qmul.ac.uk).

### Materials availability

Cell lines generated in this study (MSTOR) will be shared by the lead contact upon request.

### Data and code availability

All data reported in this paper will be shared by the lead contact upon request.

## Acknowledgments

We thank the BCI Animal Technical Service (ATS) for their help with the animal work. We thank all members of the Martin lab for helpful discussions. This work was supported by funding from the 10.13039/501100000351British Lung Foundation (MESO15-12) and Polaris Pharmaceuticals. All research conducted within the 10.13039/501100019154CRUK Barts Centre was supported by an infrastructure grant from 10.13039/501100000289CRUK (C355/A25137).

## Author contributions

Conceptualization: P.S. and S.A.M.; data curation: J.C. and M.F.; formal analysis and investigation: J.C., M.F., V.M., and K.B.; methodology: J.C., M.F., and V.M.; resources: P.S. and J.B.; funding acquisition: P.S. and S.A.M.; writing: J.C., M.F., V.M., K.B., J.B., P.S., and S.A.M.

## Declaration of interests

P.S. has received funding research support from Polaris Pharmaceuticals Inc.; J.B. is an employee of Polaris Pharmaceuticals Inc.

## STAR★Methods

### Key resources table

**Table:** 

REAGENT or RESOURCE	SOURCE	IDENTIFIER
**Antibodies**		

anti-ASS1	ATLAS antibodies	HPA02093; RRID: AB_1845118
anti-Vinculin	Cell Signaling	#4650; RRID: AB_10559207

**Chemicals, peptides, and recombinant proteins**		

ADI-PEG20	Polaris Pharmaceuticals, Inc	
Difluoromethylornithine (DFMO)	Santa Cruz	CAS 70052-12-9
4-Methylcyclohexylamine (4MCHA)	Sigma	CAS 6321-23-9
MDL72527	Insight Biotechnology	CAS. 93565-01-6
N1,N11-diethylnorspermine (DenSpm)	Bio Techne	CAS 121749-39-1
N1-Guanyl-1,7-Diaminoheptane (GC7)	Sigma	CAS 150333-69-0
Ciclopirox (CPX)	Bio Techne	CAS 29342-05-0
Devimistat (CPI-613)	Selleckchem	CAS 95809-78-2

**Critical commercial assay**		

CellTiter-Glo Luminescence Assay	Promega	G7570

**Experimental models: Cell lines**		

Ju77 (epithelioid-like MPM)	Merck	41106514
H28 (sarcomatoid MPM)	ATCC	CRL-5820
H226 (epithelioid-like MPM)	ATCC	CRL-5826
MSTO (biphasic MPM)	ATCC	CRL-2081

**Experimental models: Organisms/strains**		

Mouse: NOD.CB17*-Prkdc*^*scid*^/NCrCrl	Charles-River Laboratories	CHRL: 394

**Software and algorithms**		

GraphPad Prism	GraphPad	https://www.graphpad.com/scientific-software/prism/

### Experimental models

#### Cell lines

Cell lines were routinely grown in RPMI-1640 media (Sigma) supplemented with 10% fetal calf serum (FBS; Invitrogen) and 100U/ml penicillin and 100 μg/mL streptomycin at 37°C/5% CO_2_. To generate MSTOR (ADI-PEG20 resistant cells), 1 × 10^6^ MSTO cells were incubated with increasing concentrations of ADI-PEG20 (10 ng/mL - 1 μg/mL) until resistant cells appeared. The resistant cells were maintained in 1000 ng/mL of ADI-PEG20 for 2 weeks, following which the concentration of ADI-PEG20 was reduced to 100 ng/mL to enable cell proliferation. Following single cell selection, MSTOR colonies were expanded and maintained in RPMI medium supplemented with 1 μg/mL ADI-PEG20. Ju77R cells were generated as previously described.^15^

#### *In vivo* animal model experiments

MSTO cells (1.6 × 10^6^ cells) resuspended in PBS, were injected subcutaneously into the flanks of adult (∼7 weeks old) male NOD-SCID mice (Charles-River Laboratories). When the tumors were measurable, the mice were treated 2 times a week by interperitoneal (IP) injection with either vehicle (PBS), 5IU ADI-PEG20, 10 mg/kg GC7 alone or in combination. Tumors were measured three times weekly. The mice were sacrificed in case of sickness or when the tumors reached 1.25 cm^2^. All animal procedures were carried out as per the Animals Scientific Procedures Act 1986, under the Home Office approval licence (PP9448177).

### Method details

#### Cell viability assays

Cells were seeded in 96-well plates (1-2 × 10^3^ cells/well) 24 h before treatment with indicated compounds serially diluted in RPMI-1640 media. After 72 h treatment, cell viability was assessed with the ATP-based, luminescence assay, CellTiter-Glo (Promega). For rescue experiments, cells were seeded in 96-well plates (1-2 × 10^3^ cells/well) 24 h before treatment with GC7 (5 μM) alone or in combination with Aconitate (250 μM) and Acetyl-CoA (250 μM). After 72 h treatment, cell viability was assessed with CellTiter-Glo.

#### Cell proliferation assays

Cells were seeded in 96-well plates (1-2 × 10^3^ cells/well) 24 h before treatment with indicated compounds serially diluted in RPMI-1640 media. Cell proliferation was visualised overtime in the IncuCyte S3 live cell imaging system (Essen Bioscience, MI USA) and images of well confluency were recorded every 2 or 3 h, up to a duration of 96 h. The IncuCyte S3TM automatically calculated well confluency overtime. Well confluency was normalised against t = 0h to obtain the proliferation rate.

#### 3D spheroid generation

Cells were plated in 96-well Ultra-Low-Attachment U-bottom plates (Greiner, Corning) at a density of 10,000 cells in 200 μL medium per well. Plates were centrifuged for 10 min at 1,000G at 4°C. After 96 h, if spheroid formation was confirmed by the appearance of a darker core (necrotic core) and defined edges, spheroids were treated with compounds as indicated and analyzed overtime using the IncuCyte S3 Live-Imaging system for 7 days. Spheroid size was normalised against their t = 0 and untreated control to determine the evolution of the spheroid area during treatment.

#### Protein analysis

Cell pellets were lysed in 20 mM Tris (pH 8), 200 mM NaCl, 1 mM EDTA, 0.5% (v/v) NP40, 10% glycerol, supplemented with protease inhibitors. For western blotting, lysates were electrophoresed on Novex precast gels (Invitrogen) and immunoblotted with antibodies. This was followed by incubation with anti-IgG-horseradish peroxidase and chemiluminescent detection (Supersignal West Pico Chemiluminescent Substrate, Pierce). Immunoblotting for Vinculin was performed as a loading control.

#### Metabolomics analysis

Cells (4 × 10^5^) were plated in six-well plates in five technical replicas per each condition. After 24 h, cells were treated with either ADI-PEG20 (500 ng/mL) or GC7 (10 μM), alone or in combination. Following 24 h of treatment, cells were incubated with ^13^C_6_-glucose (CLM-1396-5, Cambridge Isotope Laboratories) medium for 14 h. Cells were then washed three times with PBS, and metabolites were extracted using cold extraction buffer (50% methanol, 30% acetonitrile, 20% ultrapure water, 50 ng/mL HEPES) at a ratio of 1 mL extraction buffer/10^6^ cells. After 15-min incubation on methanol and dry ice, cells were placed on a shaker for 15 min using a thermal mixer at 4°C and incubated for 1 h at −20°C. Cell lysates were centrifuged, and the supernatant was collected and transferred into autosampler glass vials, which were stored at −80°C until further analysis.

LC-MS analysis was performed using a Q Exactive Hybrid Quadrupole-Orbitrap mass spectrometer coupled to a VAnquish UHPLC system (Thermo Fisher Scientific). The liquid chromatography system was fitted with a Sequant ZIC-pHILIC column (150 mm × 2.1 mm) and guard column (20 mm × 2.1 mm; Merck Millipore) and temperature maintained at 35°C. The mobile phase was composed of 10 mM ammonium carbonate and 0.1% ammonium hydroxide in water (solvent A) and acetonitrile (solvent B). The flow rate was set at 100 μL/min with the gradient described previously.^32^ The mass spectrometer was operated in full MS and polarity switching mode. The acquired spectra were analyzed using Xcalibur Qual Browser and Xcalibur Quan Browser software (Thermo Scientific).

### Quantification and statistical analysis

Unless stated otherwise, data represent standard error of the mean of at least three independent experiments. The two-tailed paired Student’s t test was applied upon analysis between two experimental conditions. A 1-way ANOVA with Holm-Sidak multiple comparison post-test analysis was applied upon analysis of the difference between three or more experimental conditions. Unless specified all results shown are the outcome of three independent biological replicate assay (*n* = 3). *p* < 0.05 was regarded as significant.
