# Supplementary material for: Overcoming resistance to arginine deprivation therapy using GC7 in pleural mesothelioma
Source: iScience. 2024 Dec 2;28(1):111525. doi: 10.1016/j.isci.2024.111525 (PMC11699351; doi:10.1016/j.isci.2024.111525)
Supplement: Document S1. Figures S1–S5 [file mmc1.pdf]

iScience, Volume *28*

Supplemental information

Overcoming resistance to arginine deprivation therapy using GC7 in pleural mesothelioma

Josephine Carpentier, Marta Freitas, Valle Morales, Katiuscia Bianchi, John Bomalaski, Peter Szlosarek, and Sarah A. Martin

SUPPLEMENTARY FIGURE 1

A

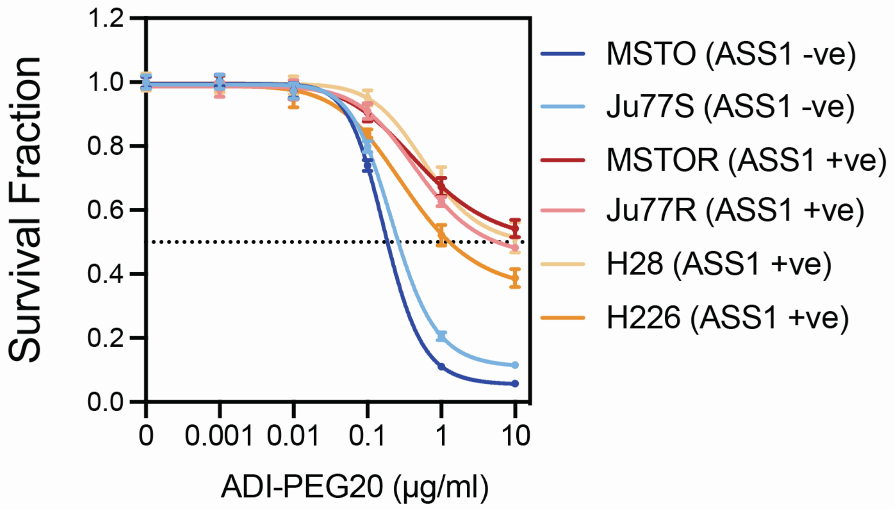

B

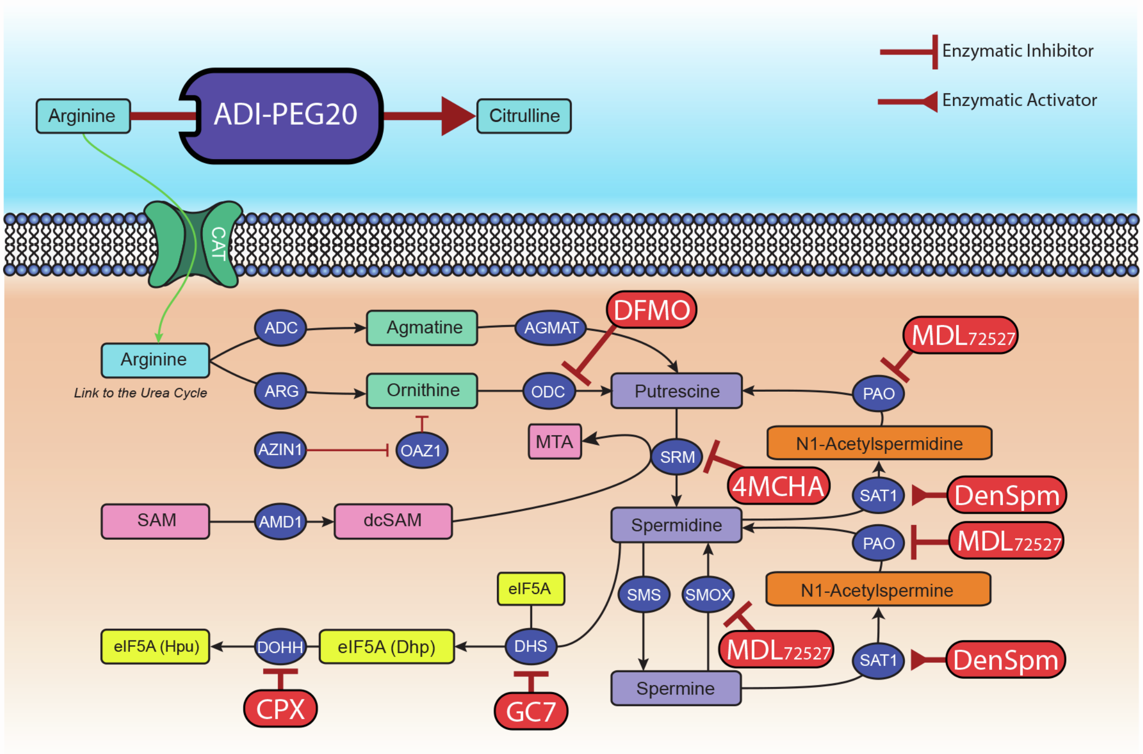

SUPPLEMENTARY FIGURE 2

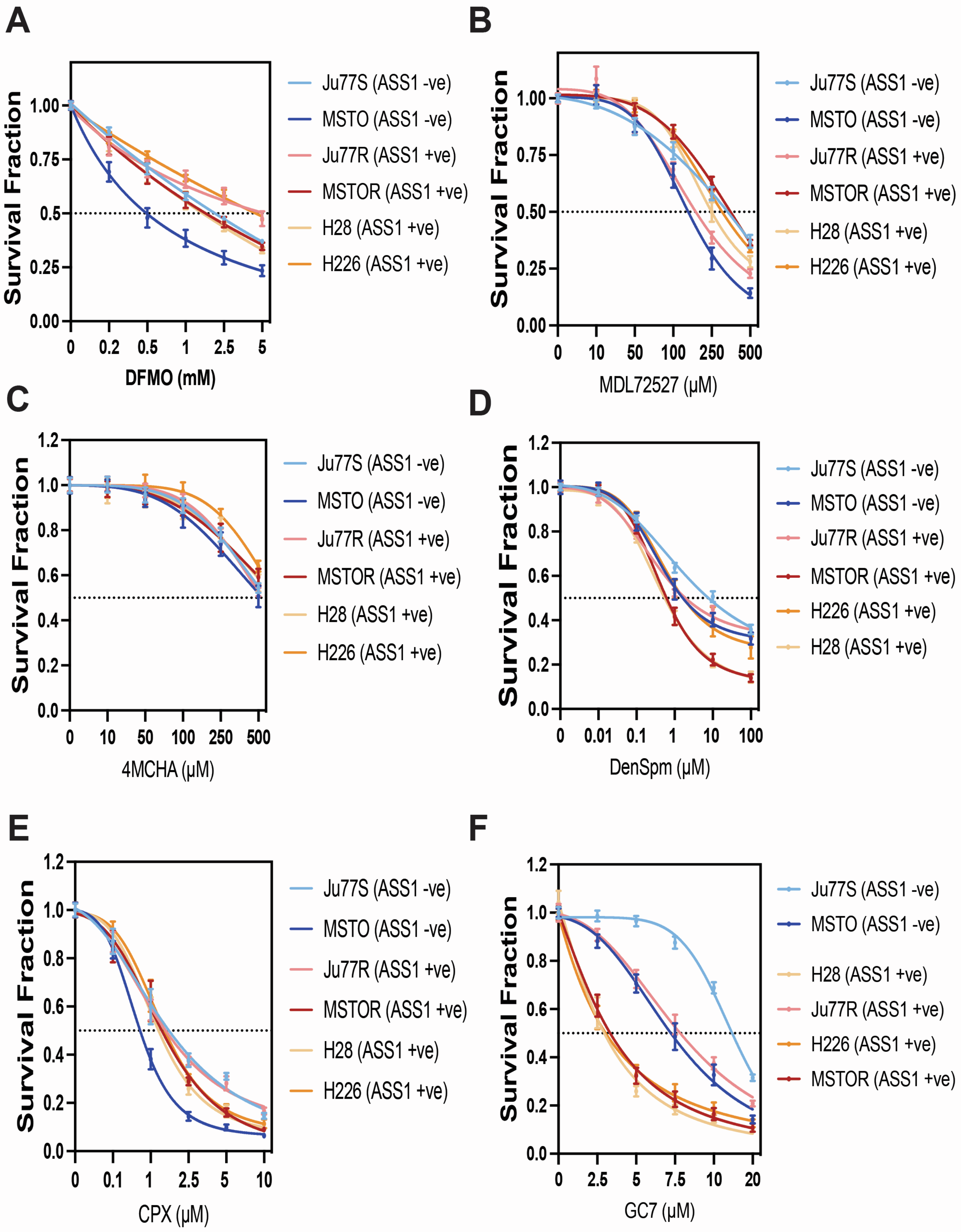

SUPPLEMENTARY FIGURE 3

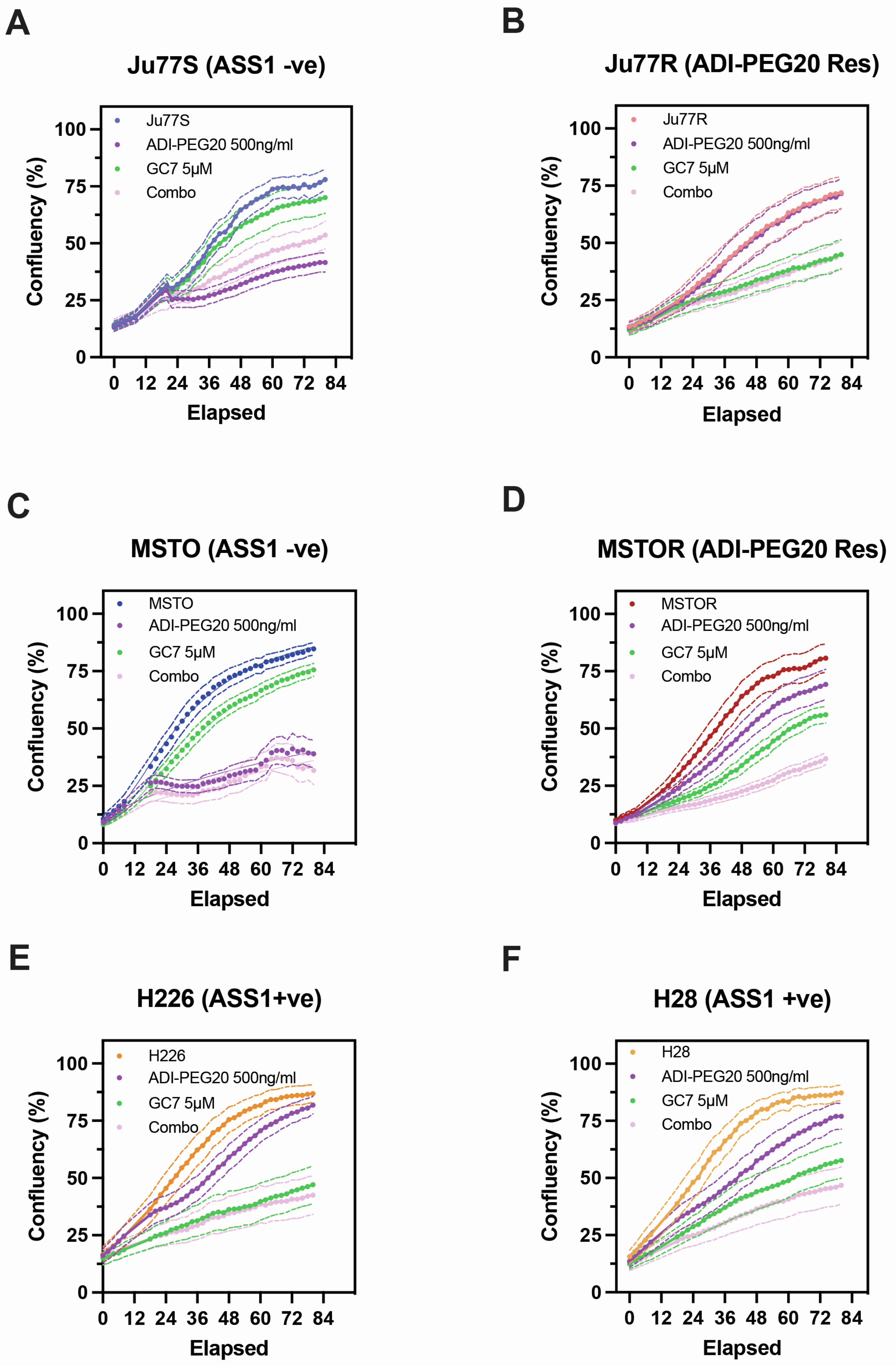

SUPPLEMENTARY FIGURE 4

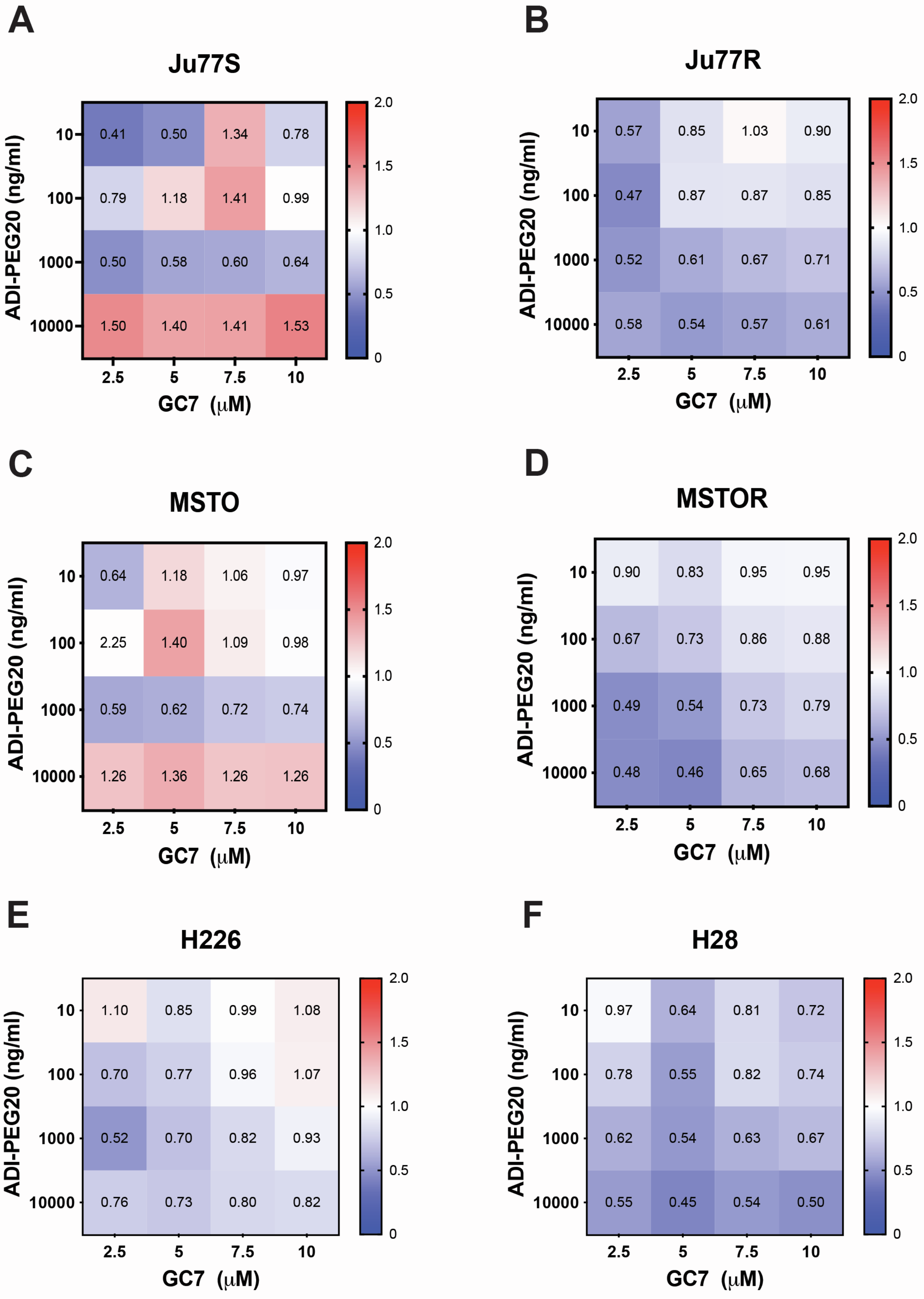

SUPPLEMENTARY FIGURE 5

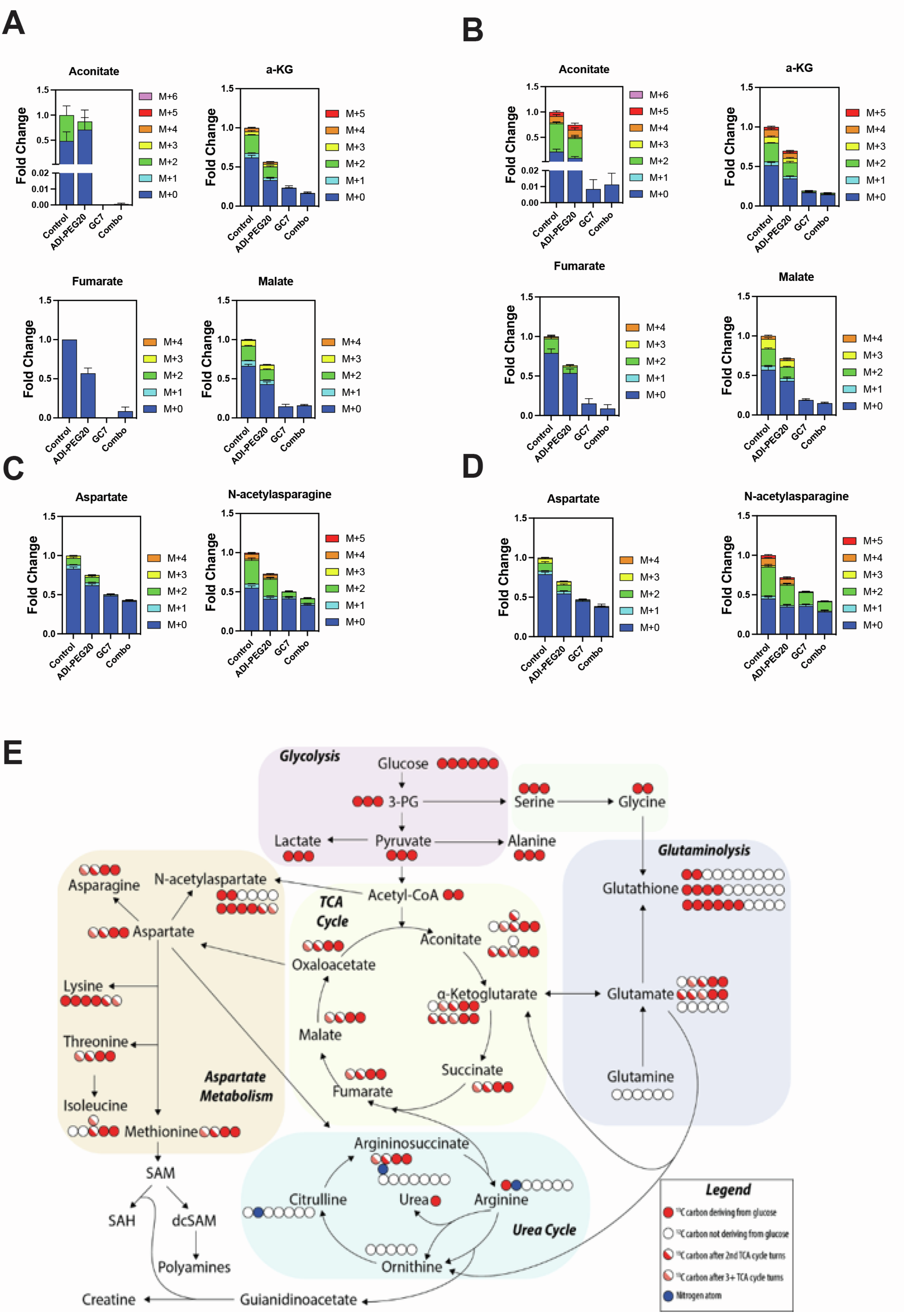

SUPPLEMENTARY FIGURE LEGENDS

SUPPLEMENTARY FIGURE 1. ASS1-deficient cells are sensitive to ADI-PEG20 treatment. (A) The ADI-PEG20 sensitive Ju77S and MSTO cells, the ADI-PEG20 resistant Ju77R and MSTOR PM cells and the ASS1-proficient H28 and H226 cells were treated with increasing concentrations of ADI-PEG20 (0, 1, 10, 100, 1000 & 10,000 ng/ml). After 72 h treatment, cell viability was measured using an ATP-based luminescence assay.

(B) Schematic representation of the enzymatic inhibitors DFMO, 4MCHA, MDL72527, GC7, CPX and the enzymatic activator DenSpm indicating where in the polyamine metabolic pathway the different compounds target. Figure S1 related to Figure 1.

SUPPLEMENTARY FIGURE 2. ADI-PEG20 resistant cells are sensitive to GC7 treatment. The ADI-PEG20 sensitive Ju77S and MSTO cells, the ADI-PEG20 resistant Ju77R and MSTOR cells and the ASS1-proficient H28 and H226 cells were treated with increasing concentrations of (A) DFMO; (B) MDL72527; (C) 4MCHA; (D) DenSpm; (E) Cicloprox and (F) GC7. After 72 h treatment, cell viability was measured using an ATP-based luminescence assay. Figure S2 related to Figure 2.

SUPPLEMENTARY FIGURE 3. Combined ADI-PEG20 and GC7 treatment decreases cell

proliferation in 3D spheroid models. The ASS1-ve Ju77S (A) and the ADI-PEG20 resistant Ju77R (B) cells, the ASS1-ve MSTO (C) cells and the ADI-PEG20 resistant MSTOR (D) cells and the ASS1-proficient H226 (E) and H28 (F) cells were treated either 500 ng/ml of ADI-PEG20 and/or 5 µM GC7. Cell proliferation was analysed using the IncuCyte live-cell imaging system and images were taken every 2 h over a period of 84 h. A confluency mask was applied to each individual image and normalised to the first image (t=0 h). Figure S3 related to Figure 3.

SUPPLEMENTARY FIGURE 4. Combination treatment of ADI-PEG20 and GC7 is synergistic in PM cells. The ASS1-ve Ju77S (A) and the ADI-PEG20 resistant Ju77R (B) cells, the ASS1-ve MSTO (C) cells and the ADI-PEG20 resistant MSTOR (D) cells and the ASS1-proficient H226 (E) and H28 (F) cells were treated with escalating concentrations of ADI-PEG20 (0, 10, 100, 1000, 10000 ng/ml), escalating concentrations of GC7 (0, 2.5, 5, 7.5, 10 µM) or a combination of all concentrations used. After 72h of treatment, cell viability was evaluated using a CTG assay. Survival fractions were normalised to luminescent values of the untreated cells. Combination indexes of the combination of ADI-PEG20 and GC7 were calculated using the CalcuSyn software. Data shown in each cell of the heat map correspond to the median combination index of the combinations as calculated by the CalcuSyn software. Figure S4 related to Figure 4.

SUPPLEMENARY FIGURE 5. GC7 treatment causes reduced TCA cycle metabolites in PM cells, MSTO (A, C) and MSTOR (B, D) cells were cultured for 24h with or without 500 ng/ml ADIPEG20 and / or 5 µM GC7 in RPMI-1640 with 2g/L 13-C labelled Glucose. Cells were

harvested for metabolite detection. Each individual metabolite isotopomers have been normalised to the total ion content of the sample. Associated isotopomers are shown as a stacked bar chart. M+0=Dark blue; M+1=Light blue; M+2=Green; M+3=Yellow; M+4=Orange; M+5=Red; M+6= Purple. (E) Schematic representation of pathways investigated with the 13C-glucose fluxomics analysis. After being cultured and fed with 13C-glucose, heavier than 12C-glucos, heavy glucose is metabolised within the cell and 13C-glucose derived carbons (red circles) are incorporated to other metabolites belonging to different pathways (glycolysis (pink), TCA cycle (yellow), glutaminolysis (blue), aspartate metabolism (orange) and urea cycle (green)). This increase of molecular weight can be detected by LC/MS to track the use of glucose. Because TCA cycle metabolites can influence several cycles, half red circles indicates glucose derived carbons after a 2nd turn and half pink circles after subsequent turns. If a Nitrogen atom is present in the middle of the carbon backbone it has been indicated in blue. Figure S5 related to Figure 5.
